# A Genetic Variant in the Promoter Region of miR-106b-25 Cluster Predict Clinical Outcome of HBV-Related Hepatocellular Carcinoma in Chinese

**DOI:** 10.1371/journal.pone.0085394

**Published:** 2014-01-08

**Authors:** Fuzhen Qi, Mingde Huang, Yun Pan, Yao Liu, Jibin Liu, Juan Wen, Kaipeng Xie, Hongbing Shen, Hongxia Ma, Yi Miao, Zhibin Hu

**Affiliations:** 1 Department of Hepatopancreatobiliary Surgery, Huai'an First People's Hospital, Nanjing Medical University, Huai'an, Jiangsu, China; 2 Department of Medical Oncology, Huai'an First People's Hospital, Nanjing Medical University, Huai'an, Jiangsu, China; 3 Department of Epidemiology and Biostatistics, Jiangsu Key Lab of Cancer Biomarkers, Prevention and Treatment, Cancer Center, School of Public Health, Nanjing Medical University, Nanjing, Jiangsu, China; 4 Department of Hepatobiliary Surgery, Nantong Tumor Hospital, Nantong, Jiangsu, China; 5 State Key Laboratory of Reproductive Medicine, Nanjing Medical University, Nanjing, China; 6 Department of General Surgery, The First Affiliated Hospital of Nanjing Medical University, Nanjing, Jiangsu, China; MOE Key Laboratory of Environment and Health, School of Public Health, Tongji Medical College, Huazhong University of Science and Technology, China

## Abstract

**Background:**

*MiR-106b-25* cluster, hosted in intron 13 of *MCM7*, may play integral roles in diverse processes including immune response, tumorigenesis and progression. A single nucleotide polymorphism (SNP), rs999885, is located in the promoter region of *MCM7*. Our previous study showed that the A to G base change of rs999885 may provide an increased risk for HCC in HBV persistent carriers by altering the expression of the *miR-106b-25* cluster. However, it is unknown whether rs999885 is associated with prognosis of intermediate or advanced HBV-related hepatocellular carcinoma (HCC) patients.

**Methods:**

The SNP, rs999885, was genotyped by using the TaqMan allelic discrimination Assay in 414 intermediate or advanced HCC patients. Log-rank test and Cox proportional hazard models were used for survival analysis.

**Results:**

The variant genotypes of rs999885 were associated with a significantly decreased risk of death for intermediate or advanced HCC [additive model: adjusted hazard ratio (HR)  = 0.76,95% confidence intervals (CI)  = 0.59–0.97]. Further stepwise regression analysis suggested that rs999885 was an independently protective factor for the prognosis of HCC in the final model (additive model: adjusted HR  = 0.72, 95% CI  = 0.56–0.91, *P* = 0.007).

**Conclusions:**

These findings indicate that the A to G base change of rs999885 may provide a protective effect on the prognosis of intermediate or advanced HCC in Chinese.

## Introduction

Primary liver cancer is the sixth most common cancer worldwide. Hepatocellular carcinoma (HCC), which represents the dominant histological type, accounts for 70–85% of primary malignancies in liver [1]. An estimated 748,300 new liver cancer cases and 695,900 cancer deaths occurred worldwide in 2008. Half of these cases and deaths were estimated to occur in China. Due to its poor prognosis and high fatality rates, the incidence and mortality rates are almost equal [2].

MiRNAs are small non-coding RNAs that function as post-transcriptional regulators, through binding to target mRNAs and negatively regulating their stability and transcriptional efficiency [3]. To date, accumulating data has revealed that a subset of miRNAs are deregulated in HCC [4]–[15]. Among those, *miR-106b-25* cluster, including *miR-106b*, *miR-93* and *miR-25*, has been investigated for its integral roles in tumorigenesis and progression of multiple cancers including HCC [9],[16]–[19]. Particularly, a study performed by Shen *et al.,* suggested that miR-106b could induce the proliferation of human hepatoma cells and play important roles in HCC progression by downregulating APC expression [20]. Studies have indicated that genetic variants of miRNAs may affect a wide spectrum of mRNA targets including oncogenes and tumor suppressor genes, by changing the function or expression of the miRNAs [21]. *MiR-106b-25* cluster is located in intron 13 of *MCM7* (minichromosome maintenance complex component 7) and our previous study [22] has showed that the A to G base change of rs999885 was associated with an increased risk of HCC in HBV persistent carriers by altering the expression of the *miR-106b-25* cluster. However, it is unclear whether rs999885 is related to the survival of HCC. Here, we explored the association between the A to G base change of rs999885 and the clinical outcome of 331 intermediate or advanced HCC patients in Chinese.

## Materials and Methods

### Participants

The study was approved by the institutional review board of Nanjing Medical University. Written informed consent was obtained from every subject. The subjects' enrollment was described previously [23], [24]. In consideration of prognostic modeling in HCC patients has a high complexity and should consider several tightly related aspects, including tumor stage, degree of liver function impairment, patient's general condition, and treatment efficacy, we use the Barcelona Clinic Liver Cancer (BCLC) Stage System to evaluate the prognosis of HCC in this study [25]. To construct a relatively homogenous population with similar treatment and considering surgery is less genetic related, our current study was restricted to HCC patients in either intermediate (B) or advanced stage (C) without surgery. We recruited 414 intermediate or advanced HCC patients from Nantong Tumor Hospital and the First Affiliated Hospital of Nanjing Medical University, Jiangsu, China. All patients were followed up prospectively every 3 months from the time of enrollment by personal or family contacts until death or last time of follow-up. As a result, a total of 331 intermediate or advanced HCC patients who had complete follow-ups and clinical information were enrolled in our study with the response rate was 80.0%. The maximum follow-up time (MFT) for the 331 patients involved in the present study was 60.7 months (last follow-up in January 2013) and the median survival time (MST) was 14.5 months.

### Serological testing

HBsAg, anti-HBs, anti-HBc and anti-HCV were detected by the enzyme-linked immunosorbent assay (Kehua Bio-engineering Co., Ltd., Shanghai, China) in the serum following the manufacturer's instructions as described previously [23].

### SNP genotyping

Genomic DNA was extracted from leukocyte pellets by traditional proteinase K digestion, followed by phenol-chloroform extraction and ethanol precipitation. The SNP, rs999885 A>G was genotyped by the TaqMan allelic discrimination Assay on an ABI 7900 system (Applied Biosystems) as described previously [22]. The polymorphism was successfully genotyped with a call rate 99.40% and also was consistent with Hardy-Weinberg equilibrium (HWE) (*P* = 0.17). A total of 10% random samples were reciprocally tested on the TaqMan assay, and the reproducibility was 100%.

### Statistical methods

Survival time was calculated from the date of HCC diagnosis to the date of patients dead or the last time of follow-up. Hardy–Weinberg equilibrium was assessed within patients by using a goodness-of-fit χ^2^ test. Median survival time (MST) was calculated, and mean survival time was presented when the MST could not be calculated. Log-rank test was used to compare the survival time in different subgroups categorized by patient characteristics, clinical features and genotypes. Univariate and multivariate Cox proportional hazard regression analysis were performed to estimate the crude or adjusted hazard ratio (HR) and their 95% confidence intervals (CI), with adjustment of age, gender, smoking status, drinking status, BCLC stage, and chemotherapy or TACE (transcatheter hepatic arterial chemoembolization) status. The Chi-square-based *Q* test was applied to test the heterogeneity of associations between subgroups. The significance level was set at *P*<0.05 and *P* values are given for two-sided tests. Statistical Analysis System software (version 9.1.3; SAS Institute, Cary, NC) was used to carry out above analyses and survSNP package in R (version 2.15.1) was used to calculate the adequate sample size in survival analyses.

## Results

### Patient characteristics and clinical features

The demographic characteristics and clinical information for the 331 HCC patients at stage B or C recruited in the study were described previously [24]. The median age was 53.0 years. Of the 331 patients, 284 (85.8%) were male, 211 (63.7%) were smokers, 204 (61.6%) were drinkers, 304 (91.8%) patients were at intermediate stage (B), and 240 (72.5%) received either chemotherapy or TACE therapy. For the 331 HCC patients in stage B or C in our survival analysis, 258 died from HCC, and 2 died from other causes during a period of up to 60.7 months of follow-up. Drinking status and chemotherapy or TACE were significantly associated with the survival time (log-rank *P* = 0.006 and <0.001 for drinking status and chemotherapy or TACE status, respectively). The patients who consumed alcohol had a higher risk of death than those who never drink (HR  = 1.43, 95%CI  = 1.11–1.84). Compared with patients without chemotherapy and TACE (MST  = 3.4 months), those with chemotherapy or TACE (MST  = 16.8 months) had a 61% significantly decreased risk of death (HR  = 0.39, 95%CI  = 0.29–0.51).

### Genotypes of *miR-106b-25* and HCC patients' survival

Log-rank test and Cox regression analyses were used to examine the association of *miR-106b-25* rs999885 and HCC survival in different genetic models. As shown in Table 1, the patients carrying AG/GG genotypes of rs999885 demonstrated a longer survival time(MST, 16.2 months) compared to patients with AA genotype (MST, 13.3 months). Then the multivariate Cox regression analysis showed that G allele of rs999885 was significantly associated with a better survival of HCC with adjustment of age, gender, smoking status, drinking status, BCLC stage, and chemotherapy or TACE status (additive model: adjusted HR  = 0.76, 95% CI  = 0.59–0.97, *P* = 0.026).

**Table 1:** Genotypes of *miR-106b-25* rs999885 and HCC patients' survival.

Genotype	Total	Deaths	MST (mo)	Crude HR (95% CI)	Adjusted HR (95% CI)^a^	*P* a
rs999885 (A>G)	N = 329	N = 256				
AA	223	178	13.3	1	1	
AG	100	74	16.0	0.83 (0.63–1.09)	0.79 (0.60–1.05)	0.103
GG	6	4	27.2	0.56 (0.21–1.52)	0.41 (0.15–1.13)	0.085
AG/GG	106	78	16.2	0.81 (0.62–1.06)	0.76 (0.58–1.00)	0.051
Additive model				0.81 (0.64–1.04)	0.76 (0.59–0.97)	0.026

MAF, minor allele frequency; HWE, Hardy-Weinberg equilibrium; MST, median survival time; HR, hazard ratio; CI, confidence interval.

^a^ Adjusted for age, gender, smoking status, drinking status, BCLC stage, and chemotherapy or TACE status.

### Stratified analysis of *miR-106b-25* rs999885 and HCC survival

The association between rs999885 and HCC survival were further evaluated by stratification analyses of age, gender, smoking status, drinking status, and chemotherapy or TACE. As shown in Table 2, the protective effect of G allele of rs999885 on HCC survival were still significant in patients younger than 53 years, males, smokers, drinkers, and in patients with chemotherapy or TACE. However, no significant heterogeneity was observed between different subgroups (*P*>0.05).

**Table 2:** Stratified analysis of rs999885 genotypes and HCC survival.

Variables	rs999885 (Patients/Deaths)			Adjusted HR (95% CI)^a^	*P* for heterogeneity
	AA	AG	GG		
**Age**					
< = 53	118/97	51/38	3/2	0.67 (0.47–0.94)	0.585
>53	105/81	49/36	3/2	0.77 (0.54–1.11)	
**Gender**					
Male	191/152	86/64	6/4	0.72 (0.55–0.93)	0.441
Female	32/26	14/10	NA	0.99 (0.46–2.13)	
**Smoking status**					
No	81/62	35/25	2/1	0.92 (0.60–1.43)	0.264
Yes	142/116	65/49	4/3	0.68 (0.50–0.92)	
**Drinking status**					
No	87/67	37/25	1/0	0.88 (0.56–1.39)	0.408
Yes	136/111	63/49	5/4	0.70 (0.52–0.94)	
**Chemotherapy or TACE**					
No	55/48	32/25	3/3	0.84 (0.55–1.29)	0.462
Yes	168/130	68/49	3/1	0.69 (0.51–0.94)	

TACE, transcatheter hepatic arterial chemoembolization; HR, hazard ratio; CI, confidence interval.

^a^ Adjusted for age, gender, smoking and drinking status, BCLC stage, and chemotherapy or TACE status (excluded the stratified factor in each stratum).

### Step Cox regression analysis on HCC survival

To get insight into the effects of demographic characteristics, clinical features, and *miR-106b-25* rs999885 on HCC survival, we performed stepwise Cox proportional hazard analysis. As shown in Table 3, four variables (chemotherapy or TACE status, age, drinking status, *miR-106b-25* rs999885) were remained in the regression model with a significance level of 0.050 for entering and 0.051 for removing a variable (*P*<0.001 for chemotherapy or TACE status; *P* = 0.002, 0.004 and 0.007 for age, drinking status, and *miR-106b-25* rs999885, respectively).

**Table 3:** Stepwise Cox Regression analysis on HCC survival.

Variables	β^a^	SE^b^	HR	95% CI	*P*
Chemotherapy or TACE (yes vs none)	−1.1792	0.1532	0.31	0.23–0.42	<0.001
Age(>53 vs < = 53)	−0.4318	0.1362	0.65	0.50–0.85	0.002
Drinking status (yes vs no)	0.4231	0.1327	1.53	1.18–1.98	0.004
rs999885 (additive model)	−0.3356	0.1255	0.72	0.56–0.91	0.007

TACE, transcatheter hepatic arterial chemoembolization; HR, hazard ratio; CI, confidence interval.

^a^β is the estimated parameter of the regression model.

b SE is the standard error of the regression model.

## Discussion

In this study, we investigated the association between rs999885 and the clinical outcome of HCC in a Chinese population and found that rs999885 genotypes were significantly associated with the overall survival of HCC patients at intermediate or advanced stage, suggesting that the A to G base change of rs999885 may serve as a prognostic marker for the survival of HCC patients.

MiR-106b-25 has been reported to be up-regulated in several cancers, including head and neck squamous cell carcinoma [26], esophageal adenocarcinoma [27], gastric cancer [28], osteosarcoma [29] and HCC [10]. Specially, Landgraf P and his colleages [30] found that the expression levels of miR-106b, miR-93 and miR-25 were higher in liver cancer cell lines than those in normal liver cells by sequencing analysis. Accumulating data has showed that *miR-106b-25* cluster plays oncogenic roles in cancers through influencing tumor growth, cell survival, and angiogenesis [21], [31], [32].

Genetic variants in miRNA genes, including pri-miRNAs, pre-miRNAs and mature miRNAs, has been shown to affect the processing and/or target selection of miRNAs, thus affecting the development and prognosis of cancer [33]–[35]. In our previous study, the rs999885 variant has been found to be associated with the chronic HBV infection and risk of HCC, which supported the role of genetic polymorphisms in the different stages of HCC development [36], [37]. In this study, we found a significant effect of *miR-106b-25* rs999885 on patients survival after the adjustment for age, smoking status, drinking status, treatment (chemotherapy or TACE) and clinical stage, suggesting ***miR-106b-25*** rs999885 is an independent prognostic factor for patients of HCC patients in stage B or C. Besides, our previous study also has indicated that the rs999885 variant could influence *miR-106b-25* expression and the expression levels of *miR-106b-25* were significantly higher in AG/GG carriers than that in AA carriers [22]. By using the web-based prediction tool, doRiNA [38], we found that miR-106b-5p (one of the miR-106b-25 cluster) can target an X-linked inhibitor of apoptosis protein (XIAP), which has been reported to exhibit elevated expression levels in hepatocellular carcinoma (HCC) and promote cell survival, metastasis and tumor recurrence [39]. Therefore, we speculate that the rs999885 variant genotypes could contribute to miR-106b-5p overexpression and then suppress cell survival, metastasis and tumor recurrence through reducing the expression levels of XIAP, thereby finally led to a good prognosis of HCC patients in our study.

Our study still has a number of limitations. To our knowledge, several studies have reported that changes in alpha-fetoprotein (AFP) and des-gamma-carboxy prothrombin (DCP) could be used as diagnostic and prognosis biomarkers for HCC [40]–[42]. However, it is regretful that we failed to collect adequate and accurate information of AFP and DCP in our study. Additionally, the relatively small sample size in our study might induce a limited statistical power. We have calculated the power by using survSNP package in R (version 2.15.1) and found that 331 subjects in our study could only achieve 44.5% power at a 0.050 significance level to detect a HR of 0.76. Therefore, a larger well-designed study and functional evaluation are warranted to confirm these findings.
